# Finite element evaluation of artery damage in deployment of polymeric stent with pre- and post-dilation

**DOI:** 10.1007/s10237-019-01194-6

**Published:** 2019-07-17

**Authors:** R. He, L. G. Zhao, V. V. Silberschmidt, Y. Liu, F. Vogt

**Affiliations:** 1 Wolfson School of Mechanical, Electrical and Manufacturing Engineering, Loughborough University, Epinal Way, Loughborough, LE11 3TU UK; 2 Medical Clinic I, University Hospital Aachen, Pauwelsstrasse 30, 52074 Aachen, Germany

**Keywords:** Artery damage, Hyperelastic damage model, Pre/post-dilation, Stent deployment, Plaque rupture

## Abstract

Using finite element method, this paper evaluates damage in an arterial wall and plaque caused by percutaneous coronary intervention. Hyperelastic damage models, calibrated with experimental results, are used to describe stress–stretch responses of arterial layers and plaque; these models are capable to simulate softening behaviour of the tissue due to damage. Abaqus CAE is employed to create the finite element models for the artery wall (with media and adventitia layers), a symmetric uniform plaque, a bioresorbable polymeric stent and a tri-folded expansion balloon. The effect of percutaneous coronary intervention on vessel damage is investigated by simulating the processes of vessel pre-dilation, stent deployment and post-stenting dilation. Energy dissipation density is used to assess the extent of damage in the tissue. Softening of the plaque and the artery, due to the pre-dilation-induced damage, can facilitate the subsequent stent deployment process. The plaque and the artery experienced heterogeneous damage behaviour after the stent deployment, caused by non-uniform deformation. The post-stenting dilation was effective to achieve a full expansion of the stent, but caused additional damage to the artery. The continuous and discontinuous damage models yielded similar results in the percutaneous coronary intervention simulations, while the incorporation of plaque rupture affected the simulated outcomes of stent deployment. The computational evaluation of the artery damage can be potentially used to assess the risk of in-stent restenosis after percutaneous coronary intervention.

## Introduction

Percutaneous coronary intervention (PCI) is a prevalent treatment of atherosclerosis to restore a normal blood flow in a coronary artery. In this stenting procedure, a balloon is generally used to inflate a stent, positioned in the diseased part of the artery, to open the blocked blood vessel. In patients with severe stenosis, pre-dilation needs to be performed before stenting in order to clear the path for easier positioning of a balloon and a stent as well as to facilitate the expansion of the vessel. On the other hand, post-dilation could be also required, if the stent is not fully expanded, in order to improve its expansion. However, mechanical stretching of the vessel induced by the PCI procedure could increase the risks of vessel injury and even rupture of the plaque and dissection of the arterial wall. The associated tissue damage can activate an inflammatory reaction of the vessel, leading to the development of in-stent restenosis (ISR) (i.e. a graduate re-narrowing of the artery), one of the major concerns in stenting.

Stents used in PCI are commonly manufactured from metals and polymers. Bare-metal stents were first developed to treat acute vessel occlusions associated with plain balloon angioplasty but can cause a high ISR rate of 20–30% (Foerst et al. 2013; Iqbal et al. 2013). To overcome this problem, drug-eluting stents were developed and proved effective in battling restenosis by inhibiting neointimal hyperplasia due to proliferation of smooth muscle cells. However, the use of metal stents is associated with some undesirable clinical complications, including late stent thrombosis, chronic inflammation, angiogenesis, negative vessel remodelling and obstruction of side branches due to the presence of permanent implants. To eliminate the potential drawbacks of metal stents, bioresorbable vascular scaffolds, made of biodegradable polymers, were recently developed to treat stenotic arteries, with complete resorption over 2–3 years. Due to their weaker mechanical properties, the polymer stents usually require pre- and post-dilations to facilitate their deployment. To understand the mechanical performance of polymer stents, some modelling work was attempted to simulate their expansion and interaction with an artery during deployment. Welch et al. (2008) performed finite element (FE) analyses of the deployment of a bioresorbable polymer stent and observed non-uniform stresses in the vessel wall exerted by stent expansion. Bobel et al. (2015) undertook comparative FE simulations of three polymeric stents with different designs; these stents demonstrated radial strength and flexibility comparable to those of mellitic ones. Schiavone et al. (2016) simulated the mechanical interaction between a polymeric stent and a blood vessel during deployment. Significantly, lower stresses, compared to those for a metal one, were found in both artery and plaque expanded with the polymer stent, which were clinically beneficial. However, these modelling work did not consider pre- and post-dilations, which are normally required for the deployment of polymer stents.

The purpose of pre- and post-dilations is to achieve a larger lumen diameter for the diseased part of the vessel after stenting, inevitably leading to damage in the plaque and the artery. Mechanical testing has been carried out to study the damage of the arterial wall and the plaque, using either animal or human tissues. Peña et al. (2009) presented experimental stress–stretch data for a pig aorta subjected to uniaxial cyclic loading, and stress-softening effect was observed for the tissue samples as a result of damage. Weisbecker et al. (2012) performed uniaxial tension tests on tissue strips, taken from 14 thoracic and 9 abdominal aortas. Their results revealed that arterial damage was primarily related to the damage of collagen fibres, the major load-bearing constituents of arteries. In parallel, models were also developed to simulate the damage behaviour of soft tissues subjected to mechanical loading, using various types of hyperelastic models. Balzani et al. (2006) simulated the overstretching of an atherosclerotic artery with the FE method, considering vessel damage and residual stresses in its wall. They noticed that the maximum damage was located at the outer boundary of the media layer, which might be caused by residual stresses between the media and the adventitia layers. Calvo et al. (2007) presented and validated a three-dimensional finite-strain anisotropic damage model for a fibrous soft tissue and applied it to simulate balloon angioplasty for coronary arteries. Damage was shown to occur in a large region of the media layer after balloon angioplasty. Conway et al. (2014) investigated the effects of different isotropic hyperelastic models for the plaque, with the consideration of calcifications, a lipid pool and the Mullins effect, on the stenting-caused deformation of the artery using FE simulations. The results indicated that arterial behaviour was dominated by the type of hyperelastic model used for the plaque. Later, they modelled the effects of pre-dilation on the mechanical response of the artery during stent deployment, considering the Mullins effect and plastic deformation for the plaque (Conway et al. 2017). It was concluded that the Mullins effect had negligible influence on the overall deformation of the artery while the impact of plastic deformation was significant. But none of the simulation work studied the damage of the arterial layers caused by stent deployment, neither the effect of post-dilation on stenting and tissue damage.

Clinical outcomes of stent deployments with pre- and/or post-dilation were also reported for polymeric stents. Rzeszutko et al. (2013) described a deployment procedure of Absorb GT1™ Bioresorbable Vascular Scaffold (BVS) (Abbott Vascular, USA), including the pre-dilation and the post-dilation procedures. They indicated that it was necessary to appropriately prepare the plaque by effective pre-dilation (with semi- or non-compliant balloon) to reduce the residual stenosis below 40%, while post-dilation (with an adequately sized non-compliant balloon) should be performed in the case of evident residual stenosis. Brugaletta et al. (2015) compared the one-year outcomes for Absorb BVS and Everolimus-eluting metallic stent for patients in a clinical trial. Their results demonstrated that BVS was associated with a higher use of pre- and post-dilations compared to the metallic stent. In addition, De Ribamar Costa Jr. et al. (2015) found no negative angiographic or clinical outcomes associated with post-dilation for Absorb BVS and suggested that the post-dilation procedure should be performed when necessary. Therefore, pre- and post-dilations were proved clinically safe and are highly recommended for stent implantation, especially for BVSs made of biodegradable polymers.

To the authors’ best knowledge, computational studies regarding the contributions of pre/post-dilations to stent deployment and associated vessel damage were not reported for BVSs in the literature before. Therefore, the aim of this paper is to investigate the effects of pre/post-dilations on the deployment of a bioresorbable polymeric stent, with a focus on assessing the damage to the arterial wall caused by the PCI procedure. Appropriate damage models are introduced to describe the softening effect of the plaque and vessel wall. FE simulations of the PCI procedure are carried out for the Absorb BVS with or without pre/post-dilation. In particular, different types of damage models for the plaque are also explored to study their effect on the simulation results.

## Constitutive models

### Hyperelastic damage model

Mechanical response of biological tissue is typically hyperelastic with stress-softening effect. The stress softening of a hyperelastic material can be described by the Mullins effect, for which damage accumulates when the load increases beyond its peak value in the preceding deformation history (Peña et al. 2009). In this section, hyperelastic models with damage are introduced to describe the damage of the plaque and arterial layers, including the calibration of model parameters against experimental measurements in the literature.

#### Damage model for plaque

The first-order Ogden hyperelastic model (Ogden 1972) was used to describe the constitutive behaviour of the plaque (assumed to be isotropic),

1 $$ \psi = \psi_{\text{vol}} + \bar{\psi } = \frac{1}{D}\left( {J - 1} \right)^{2} + \frac{2\mu }{{\alpha^{2} }}\left( {\bar{\lambda }_{1}^{\alpha } + \bar{\lambda }_{2}^{\alpha } + \bar{\lambda }_{3}^{\alpha } - 3} \right), $$

where $\psi_{\text{vol}}$ and $\bar{\psi }$ are the volumetric and isochoric parts of strain energy, respectively, $\mu$, $\alpha$ and $D$ are the material parameters, $J$ is the volumetric ratio, $\lambda_{1}$, $\lambda_{2}$ and $\lambda_{3}$ are the principal stretches (ratios) and the bar denotes isochoric values. According to the classical linear theory of isotropic elasticity, $\mu$ and $\alpha$ are related to the conventional shear modulus $G$ by $2G = \mu \alpha$, and $D$ represents the compressibility of the material.

To describe the Mullins effect, Ogden and Roxburgh (1999) proposed a pseudo-elastic damage model defined as

2 $$ \begin{array}{*{20}c} {\psi = \psi_{\text{vol}} + \eta_{\text{dis}} \bar{\psi }^{0} + \phi_{\text{dis}} (\eta_{\text{dis}} ),} \\ \end{array} $$

with

3 $$ \eta_{\text{dis}} = 1 - \frac{1}{r}{\text{erf}}\left[ {\frac{1}{m}\left( {\bar{\psi }^{{\rm max} } - \bar{\psi }^{0} } \right)} \right], $$

where $\eta_{\text{dis}} \in (0,1]$ is the discontinuous damage variable, the superscript 0 refers to the primary loading path, $\phi_{\text{dis}}$ is the (smooth) discontinuous damage function, *m* and *r* are the positive parameters, erf() is the error function and $\bar{\psi }^{{\rm max} }$ denotes the maximum free energy in the deformation history. Here, $\bar{\psi }^{0} \le \bar{\psi }^{{\rm max} }$ ensures $\eta_{\text{dis}} \le 1$, while to ensure $\eta_{\text{dis}} > 0$, *r* should be greater than 1. When $\bar{\psi }^{0} = \bar{\psi }^{{\rm max} }$, i.e. $\eta_{\text{dis}} = 1$, the material is under its primary loading. Physically, *m* controls the extent of the damage relative to the deformation. When the value of *m* is small, the damage is significant for small strain, and the material response in the small-strain region does not change markedly after the subsequent primary loading. When the value of *m* is large, the level of damage is relatively low for small strain, but the material’s response in the small-strain region changes considerably after the subsequent primary loading. On the contrary, *r* controls the extent of the damage relative to the virgin state. A larger value of *r* means less damage caused to the material.

#### Damage model for arterial layers

The so-called *HGO*-*C* model for anisotropic hyperelastic materials was used to describe the constitutive behaviour of the arterial layers (Holzapfel et al. 2000; Gasser et al. 2006; Dassault Systèmes 2017). The strain energy function of the HGO-C model is

4 $$ \psi = \psi_{\text{vol}} + \bar{\psi }_{m} + \mathop \sum \limits_{\alpha = 1}^{N} \bar{\psi }_{f,\alpha } = \frac{1}{D}\left( {\frac{{J^{2} - 1}}{2} - \ln J} \right) + C_{10} \left( {\bar{I}_{1} - 3} \right) + \frac{{k_{1} }}{{2k_{2} }}\mathop \sum \limits_{\alpha = 1}^{N} \left[ {\exp \left( {k_{2}\langle \bar{E}_{\alpha }^{2}\rangle } \right) - 1} \right], $$

with

5 $$ \bar{E}_{\alpha } \mathop = \limits^{\text{def}} \kappa \left( {\bar{I}_{1} - 3} \right) + \left( {1 - 3\kappa } \right)\left[ {\bar{I}_{4(\alpha \alpha )} - 1} \right], $$

where $\bar{\psi }_{m}$ and $\bar{\psi }_{f,\alpha }$ are the isochoric energy stored in non-collagenous matrix and collagen fibres, respectively, $C_{10}$ and $k_{1}$ are the stress-like parameters, $k_{2}$ is the dimensionless parameter, $\langle \rangle$ stands for the Macaulay brackets, $\kappa  (0 \le \kappa \le 1/3)$ is the temperature-dependent material parameter describing the level of dispersion in the fibre directions, *N* is the number of families of fibres (*N* ≤ 3), $I_{1}$ is the first principal invariant of tensor **C** (i.e. $I_{1} = {\text{tr}}{\mathbf{C}} = \lambda_{1}^{2} + \lambda_{2}^{2} + \lambda_{3}^{2}$) and its isochoric part is $\bar{I}_{1} = J^{ - 2/3} I_{1}$, and $\bar{I}_{4(\alpha \alpha )}$ is the invariants of ${\bar{\mathbf{C}}}$ and ${\mathbf{a}}_{\alpha }$ ($\bar{I}_{4(\alpha \alpha )} = {\mathbf{a}}_{\alpha } \cdot {\bar{\mathbf{C}}\mathbf{a}}_{\alpha }$; ${\mathbf{a}}_{\alpha }$ is the unit vectors used to define the mean directions of the fibres in the reference configuration). When $\kappa = 0$, the fibres are perfectly aligned in the same direction without dispersion; when $\kappa = 1/3$, the fibres are randomly distributed, i.e. behaviour is isotropic. However, Nolan et al. (2014) found that this HGO-C model cannot be used to simulate an anisotropic compressible hyperelastic material correctly, because $\bar{\psi }_{f,\alpha }$ in Eq. (4), which is the isochoric anisotropic term, cannot fully represent anisotropic contributions to the stress tensor for slightly compressible materials due to its insensitivity to volumetric deformation. Therefore, the anisotropic term should be modified by using the full invariant $I_{4(\alpha \alpha )} = J^{2/3} \bar{I}_{4(\alpha \alpha )}$ to account for material’s compressibility, and this modified HGO-C model is referred as the *modified anisotropic* (MA) model. By applying such modification, Eq. (5) becomes

6 $$ E_{\alpha } \mathop = \limits^{\text{def}} \kappa \left( {\bar{I}_{1} - 3} \right) + \left( {1 - 3\kappa } \right)\left[ {I_{4(\alpha \alpha )} - 1} \right]. $$

To describe the Mullins effect as well as permanent deformation, Fereidoonnezhad et al. (2016) modified the free energy function as

7 $$ \psi = \psi_{\text{vol}} + \bar{\psi }_{m}^{0} + \mathop \sum \limits_{\alpha = 1}^{N} \left[ {\eta_{f,\alpha } \psi_{f,\alpha }^{0} + \phi_{f,\alpha } \left( {\eta_{f,\alpha } } \right)} \right] - \left[ {\left( {1 - \eta_{in} } \right)\psi_{in}^{*} \left( {I_{i}^{*} } \right) + \phi_{in} \left( {\eta_{in} } \right)} \right], $$

where $\eta_{f,\alpha }$ and $\phi_{f,\alpha } (\eta_{f,\alpha } )$, $\eta_{in}$ and $\phi_{in} (\eta_{in} )$ are the damage variables and damage functions for the Mullins effect and permanent deformation, respectively and $\psi_{in}^{*} \left( {I_{i}^{*} } \right)$ is the (anisotropic) inelastic energy dissipation. The expressions for $\eta_{f,\alpha }$ and $\eta_{in}$ are given as

8 $$ \eta_{f,\alpha } = 1 - \frac{1}{{r_{f} }}{\text{erf}}\left[ {\frac{1}{{m_{f} }}\left( {\psi_{f,\alpha }^{{\rm max} } - \psi_{f,\alpha }^{0} } \right)} \right], $$

9 $$ \eta_{in} = \frac{{\tanh \left[ {\frac{{\bar{\psi }_{m}^{0} + \psi_{f,\alpha }^{0} }}{{\left( {\bar{\psi }_{m} + \psi_{f,\alpha } } \right)^{{\rm max} } }}} \right]^{{m_{2} }} }}{\tanh 1}. $$

In Eq. (7), $\psi_{in}^{*} \left( {I_{i}^{*} } \right)$ is assumed to be of the same form as the elastic strain energy function, but with different model parameters, and expressed as

10 $$ \psi_{in}^{*} \left( {I_{i}^{*} } \right) = C_{10}^{*} \left( {\bar{I}_{1}^{*} - 3} \right) + \frac{{k_{1}^{*} }}{{2k_{2}^{*} }}\mathop \sum \limits_{\alpha = 1}^{N} \left[ {\exp \left( {k_{2}^{*} \langle E_{\alpha }^{*2} \rangle} \right) - 1} \right], $$

with

11 $$ E_{\alpha }^{*} = \kappa^{*} \left( {\bar{I}_{1}^{*} - 3} \right) + \left( {1 - 3\kappa^{*} } \right)\left[ {I_{4(\alpha \alpha )}^{*} - 1} \right], $$

where $C_{10}^{*}$, $k_{1}^{*}$, $k_{2}^{*}$ and $\kappa^{*}$ are the material parameters for permanent deformation and $\bar{I}_{1}^{*}$ and $I_{4(\alpha \alpha )}^{ *}$ are the strain invariants at the peak deformation of the loading history, i.e. when $\bar{\psi }_{m}^{0} + \psi_{f,\alpha }^{0} = \left( {\bar{\psi }_{m} + \psi_{f,\alpha } } \right)^{{\rm max} }$.

#### Calibration of damage model parameters

**Fig. 1:**
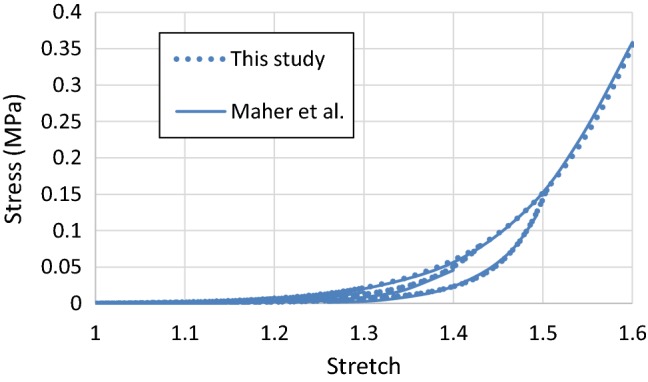
Stress–stretch responses of plaque simulated with Ogden model with Mullins effect in comparison with experimental data for echolucent plaque in Maher et al. (2011), where the unloading/reloading occurred at a stretch level of 1.1, 1.2, 1.3, 1.4 and 1.5

**Fig. 2:**
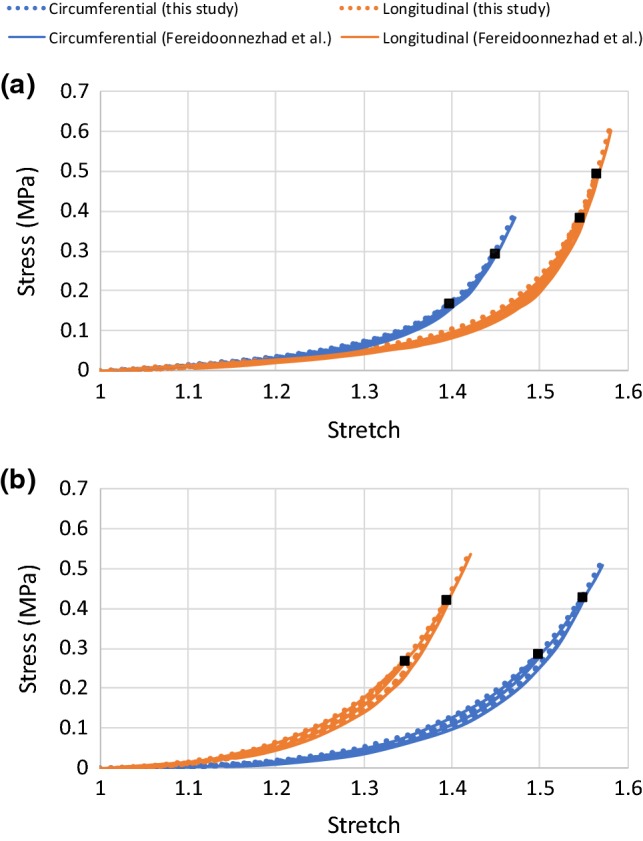
Stress–stretch responses of media (**a**) and adventitia (**b**) simulated using MA model with Mullins effect and permanent deformation, in comparison with experimental data for thoracic aortas in Fereidoonnezhad et al. (2016), where the black square symbols indicate the unloading/reloading points

The parameters of the Ogden model with Mullins effect described by Eqs. (1)–(3) were determined by fitting the experimental data for the echolucent plaque in Maher et al. (2011); the corresponding values are given in Table 1, with the density taken from Rahdert et al. (1999). The parameters of the MA model with damage, described by Eqs. (4) and (6)–(11), were determined by fitting the experimental data for thoracic aortas (Weisbecker et al. 2012; Fereidoonnezhad et al. 2016). In this study, it was assumed that there were two families of fibres, embedded symmetrically in the tangential surface of each arterial layer, and $\varphi$ represents the angle between the mean direction of fibres and the circumferential direction in the artery. The corresponding parameter values for the media and the adventitia are given in Table 2. A VUMAT subroutine, interfaced with Abaqus (Dassault Systèmes 2017), was written for the MA model with damage. The stress–stretch responses for the plaque and the arterial layers, simulated with single element models under uniaxial tensile stretch, are shown in Figs. 1 and 2, respectively, which are in good agreement with the corresponding experimental results (Maher et al. 2011; Fereidoonnezhad et al. 2016).

**Table 1:** Parameter values of Ogden model with Mullins effect for plaque

*ρ* (t/mm^3^)	$\mu$ (MPa)	$\alpha$	$D$ (MPa^−1^)	*r*	*m* (mJ/mm^3^)
1.22E − 9	0.00396803	13.8367	0.239019	1.3	0.008

**Table 2:** Parameter values of MA model with damage for arterial layers (Fereidoonnezhad et al. 2016)

Media	*ρ* (t/mm^3^)	$C_{10}$ (MPa)	$D$ (MPa^−1^)	$k_{1}$ (MPa)	$k_{2}$	$\kappa$	$\varphi$ (°)
	1.066E − 9	0.020	0.001	0.112	20.610	0.24	41.0

	$C_{10}^{ *}$ (MPa)	$k_{1}^{ *}$ (MPa)	$k_{2}^{ *}$	$\kappa^{ *}$	$r_{f}$	$m_{f}$ (MPa)	$m_{2}$
	0.000529	0.001648	0.028	0.27	3.36	0.0151	3.03

Adventitia	*ρ* (t/mm^3^)	$C_{10}$ (MPa)	$D$ (MPa^−1^)	$k_{1}$ (MPa)	$k_{2}$	$\kappa$	$\varphi$ (°)
	1.066E − 9	0.008	0.001	0.362	7.089	0.17	50.1

	$C_{10}^{ *}$ (MPa)	$k_{1}^{ *}$ (MPa)	$k_{2}^{ *}$	$\kappa^{ *}$	$r_{f}$	$m_{f}$ (MPa)	$m_{2}$
	0.000333	0.001445	0.460	0.27	2.70	0.0200	2.23

### Material models for stent and balloon

A polymeric scaffold ABSORB was considered in our simulations. The scaffold was made of PLLA, with a density of 1.4E−9 t/mm^3^, the Young’s modulus of 2200 MPa, and the Poisson’s ratio of 0.3 (Schiavone et al. 2016). Plastic behaviour of PLLA was described by the stress–strain response provided in Pauck and Reddy (2015). The compliant balloon used for stenting was assumed to be made of poly(1,8-octanediol-co-citrate) (POC), with a density of 1.1E−9 t/mm^3^, the Young’s modulus of 49.79 MPa, and the Poisson’s ratio of 0.31 (Ponkala et al. 2012). For post-dilation, either the same balloon or a non-compliant balloon was used. The non-compliant balloon was assumed to be made of polyethylene terephthalate (PET), with a density of 1.4E−9 t/mm^3^, the Young’s modulus of 2000 MPa, and the Poisson’s ratio of 0.44 (Goodfellow 2018).

## Finite element simulations

### Models of artery, plaque, stent and balloon

**Fig. 3:**
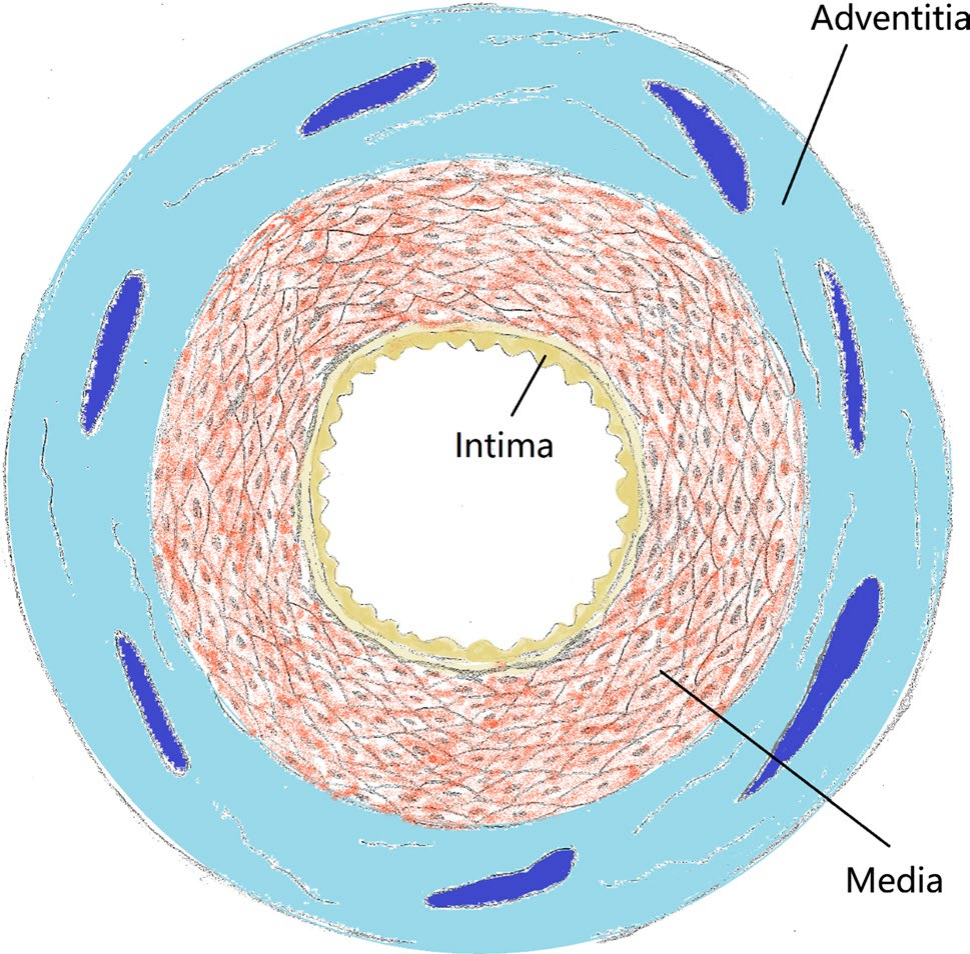
Schematic of coronary artery layers (cross-sectional view)

**Fig. 4:**
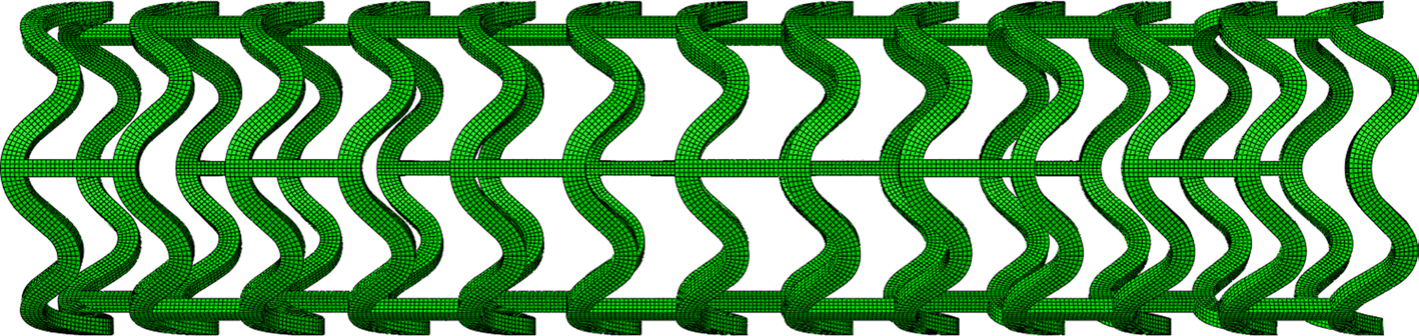
Finite element mesh for the stent

**Fig. 5:**
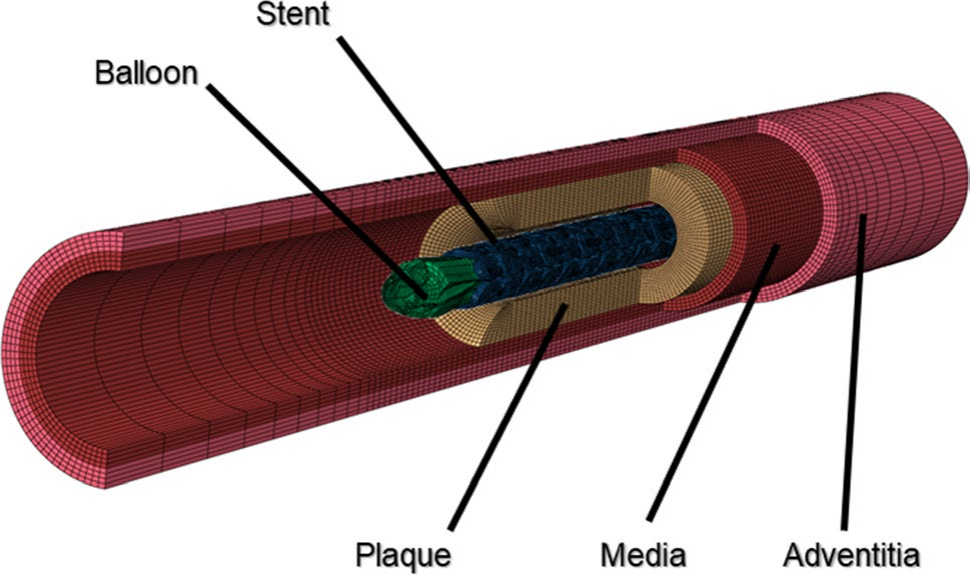
Finite element model for artery-plaque-stent-balloon assembly

For the artery, a two-layered model was created, with an inner diameter of 3 mm and a length of 40 mm reflecting a real-life case. The overall thickness of the arterial wall was 0.66 mm, including the adventitia layer of 0.34 mm and the media layer of 0.32 mm (Holzapfel et al. 2005). The extremely thin intima layer was not considered in the simulations due to its negligible contributions to artery deformation. For young healthy adults, the intima usually consists of one or two layers of endothelial cells and has a thickness of 2–4 µm as shown in Fig. 3 (Crawford et al. 2009; the intima thicknesses may increase slightly with age). In most of the existing studies, the artery was modelled with a very thick intima, which was not appropriate. In fact, the neointima (i.e. plaque) was mistaken as the intima layer. A diseased artery (i.e. with a plaque) is absent of a healthy intima layer and consequently excluded in this work. The plaque was modelled as a symmetric layer inside the artery, with a length of 10 mm and a stenosis of 50% (i.e. an inner diameter of 1.5 mm). Hexahedral elements with reduced integration (C3D8R) were used to mesh the artery and the plaque. In the radial direction, the artery was meshed with four rows of elements for each tissue layer, and the plaque was meshed with eight rows of elements. In the longitudinal direction, the element size for the artery was increasing from its middle to the both ends by using a bias control method. The stent had a length of 12.66 mm and an outer diameter of 3 mm, while the tri-folded balloon had a length of 16 mm and a diameter of 3.2 mm in fully inflated shape. The stent and the balloon models were the same as those in Qiu et al. (2018). C3D8R and M3D4R three-dimensional four-node membrane elements with reduced integration were used to mesh the stent and the balloon, respectively. The FE mesh for the stent is shown in Fig. 4. The stent-balloon assembly was first crimped by 12 rigid plates to fit into the diseased artery. The developed FE model for the artery-plaque-stent-balloon assembly is shown in Fig. 5.

### Interaction, loading and boundary conditions

The displacements of both ends of the artery were fully fixed throughout the simulations to consider the constraints imposed by the human body environment. Viscous pressure of 0.001 MPa was uniformly applied to the outer surface of the artery to avoid sudden jumps of the expansion and also to decrease the possible oscillation. A linear elastic tube was used to pre-dilate the artery before stenting. Cosinusoidal velocities with different magnitudes were used to control the pre-dilation to different maximum diameters, i.e. 2.8, 3.0 and 3.2 mm. Interaction between the artery and the tube was modelled as general hard contacts with a frictional coefficient of 0.25 (Ju et al. 2008). The hard contact in Abaqus ensures no penetration for surfaces in contact, and also there is no limit to the magnitude of contact pressure that can be transmitted when the surfaces are in contact (Dassault Systèmes 2017). The process of stent expansion in the diseased artery consisted of inflation and deflation steps. The former was performed by applying pressure on the inner surface of the balloon with both ends fully constrained. For stenting, the pressure was increased linearly from 0 to 0.6 MPa, which was within the pressure range recommended for deployment of ABSORB. As the balloon and the artery underwent large deformation during the stenting procedure, it was possible for them to have contact with all the surfaces of the stent. Consequently, the surfaces of the whole stent, including the internal, side and outer surfaces, were selected to define its contact with both the balloon and the artery in our simulations (i.e. general hard contacts with a frictional coefficient of 0.25). Subsequently, the deflation step was modelled by releasing the pressure on the inner surface of the balloon, allowing the expanded stent to recoil freely. There was still a physical contact between the balloon and the stent due to the recoiling of the stent (i.e. recovery of elastic deformation). Therefore, interactions between stent, balloon and artery were maintained in this step. For post-dilation, the pressure was chosen to be greater than or equal to the peak inflation pressure, i.e. between 0.6 and 1.0 MPa, in order to achieve an increased lumen diameter. Again, interaction between the artery and the balloon was modelled as general hard contacts with a frictional coefficient of 0.25. All simulations were carried out by using the Abaqus explicit solver (Dassault Systèmes 2017). The time parameter was chosen to be 0.1 s for each inflation and deflation step, and the time increment was of the order of 10^−7^ s throughout the simulations. All the jobs were running on the cluster of Loughborough University (UK), each using 12 cores, and took about 12 h to finish.

## Results

In the stenting simulations, the radial displacements of two nodes, in the middle section of the inner surface of the plaque, were traced to obtain the lumen diameter. The dissipation energies (density), averaged over 8 and 4 elements in the middle sections of the plaque and the arterial layers, respectively, were used to represent the damage levels in the plaque, media and adventitia layers caused by stenting.

### Effect of pre-dilation on tissue damage and stenting

**Fig. 6:**
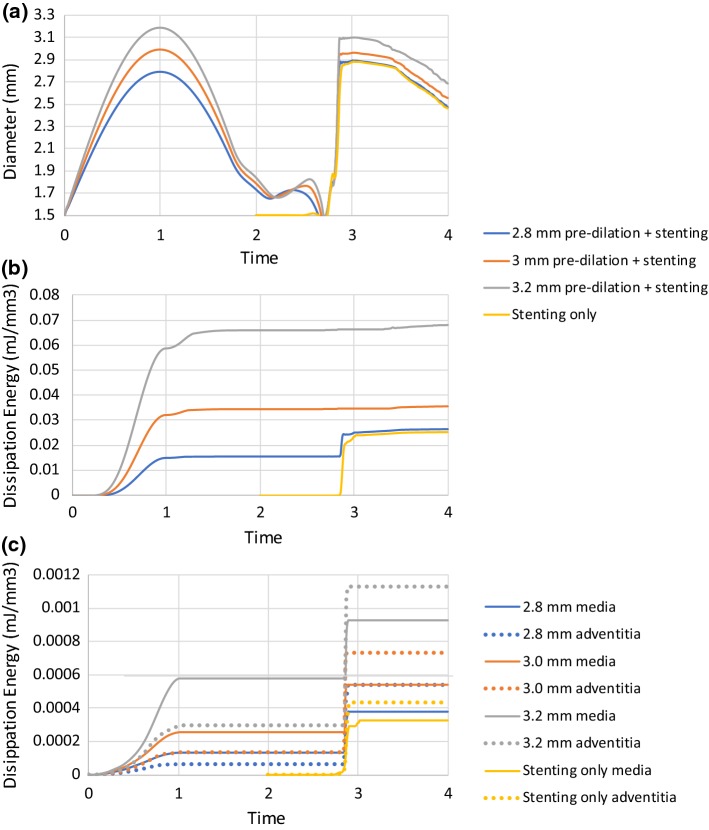
Effect of pre-dilation on tissue damage and stenting: evolutions of **a** lumen diameter; **b** dissipation energy due to damage of plaque; **c** dissipation energies due to damage of media and adventitia

The results of stenting simulated with and without pre-dilation are compared in Fig. 6. Here, the step time is normalized against the step time, for clearer presentation and more direct comparison of the results in each step. The pre-dilation, as well as stenting, consisted of both inflation and deflation steps and corresponded to periods of 0–2 and 2–4, respectively. When the artery was pre-dilated to a lumen diameter beyond 3 mm, the follow-up stenting could expand the diseased artery to a larger lumen diameter when compared to the stenting-only procedure. Further pre-dilation led to an even larger lumen diameter in the follow-up stenting step. However, such effect did not occur if the pre-dilated lumen was below 2.8 mm. The level of damage in the plaque was always much higher than that in the media and the adventitia. The damage of the plaque depended on the peak lumen diameter achieved during pre-dilation, and further damage only occurred when the previous peak lumen diameter was exceeded, while the damage of the media and the adventitia always increased with the increasing lumen diameter, even when the peak lumen diameter achieved during the stenting was less than that in the pre-dilation. Furthermore, the media suffered more damage than the adventitia during pre-dilation, while the adventitia accumulated more damage than the media during the stenting process.

### Effect of post-dilation on tissue damage and stenting

**Fig. 7:**
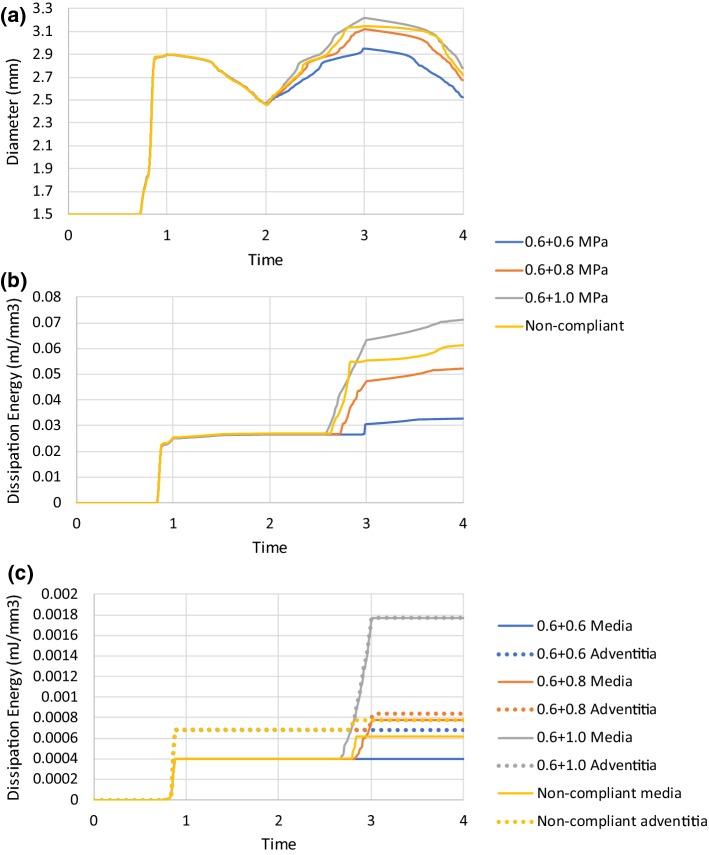
Effect of post-dilation on tissue damage and stenting: evolutions of **a** lumen diameter; **b** dissipation energy due to damage of plaque; **c** dissipation energies due to damage of media and adventitia

The effects of post-dilation on simulated stent deployment are presented in Fig. 7. The durations for stenting and post-dilation simulations were from 0 to 2 and 2 to 4, respectively. During the stenting process, the lumen diameter remained unchanged at the beginning, followed by a rapid increase and then a saturation with the increasing pressure. After the deflation, the lumen diameter experienced a gradual decrease, leading to a recoiling effect. For the post-dilations, simulated using the compliant balloon (the same as used in the stenting), the peak and final lumen diameters increased with the increase in peak pressure. Similar to the results obtained for the pre-dilation, the damage in the plaque was much higher than that in the arterial layers and increased with the increase in the lumen diameter. The damage of the arterial layers also increased with the increase in the peak lumen diameter. Also, the damage of the media was lower than that of the adventitia, similar to the simulation results with the pre-dilation. However, when the peak pressure used for the post-dilation increased, the difference of damage between the media and the adventitia decreased. This could be due to the fact that the expansion of the artery in the stenting or the post-dilation was easier than that in the pre-dilation, leading to increased deformation in the arterial layers during these stages.

**Fig. 8:**
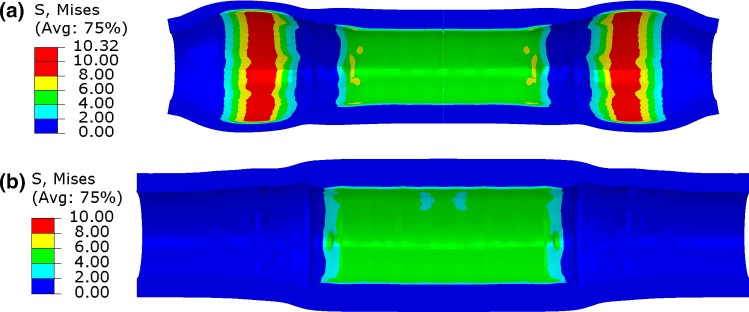
Contour plots of von Mises stress (MPa) in artery after post-dilation with **a** compliant and **b** non-compliant balloons

For the post-dilation simulated using the non-compliant balloon with a peak pressure of 1.0 MPa, the peak and final lumen diameters were smaller than those modelled with the compliant balloon with the same peak pressure, as shown in Fig. 7. This is also the case for the damage in the plaque and the arterial layers (Fig. 7). However, the stress contours at the peak pressure of post-dilation, plotted in Fig. 8, indicate a dog-boning effect of the artery for simulation using the compliant balloon. This also caused relatively higher stresses in the artery towards both ends of the plaque. In contrast, high stresses were fully contained in the plaque when a non-compliant balloon was used for post-dilation simulations.

### Effects of continuous damage behaviour of plaque

Tissue damage can be classified into discontinuous damage and continuous damage. For discontinuous damage (also known as the Mullins effect; see Sect. 2), damage accumulates only when the load exceeds the peak value in the previous deformation history, while for continuous damage, damage accumulates during the whole deformation process, irrespective of the previous loading history and path. Continuous damage is a typical mechanical behaviour of biological tissues under tension (Peña et al. 2009); however, such behaviour was not considered in the simulations of PCI. According to Miehe (1995), the continuous damage model can be similarly written as

12 $$ \psi = \psi_{\text{vol}} + \eta_{\text{con}} \bar{\psi }^{0} + \phi_{\text{con}} \left( {\eta_{\text{con}} } \right), $$

where the continuous damage variable $\eta_{\text{con}} \in (0,1]$ is assumed to be

13 $$ \eta_{\text{con}} = 1 - d\left[ {1 - \exp \left( { - \frac{\beta }{\gamma }} \right)} \right], $$

with

14 $$ \beta \left( t \right) = \mathop \smallint \limits_{0}^{t} \left| {\dot{\bar{\psi }}^{0} \left( s \right)} \right|{\text{d}}s, $$

where $d$ and $\gamma$ are the positive parameters, which ensure $\eta_{\text{con}} \le 1$, *t* is the time, and the dot denotes a derivative with respect to time. Since $\eta_{\text{con}}^{\hbox{min} } = 1 - d$, the value of *d* should be less than 1 in order to ensure $\eta_{\text{con}} > 0$. Also, $\gamma$ is related to damage saturation, and the initial condition of $\beta$ is zero, i.e. $\beta \left( 0 \right) = 0$.

**Fig. 9:**
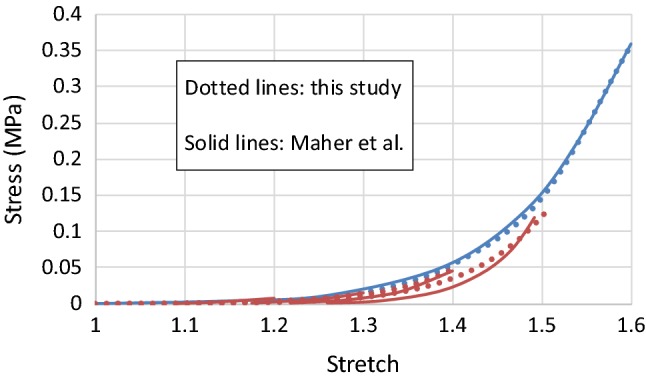
Stress–stretch responses of plaque simulated using Ogden model with continuous damage in comparison with experimental data for echolucent plaque (Maher et al. 2011) (blue lines: loading; red lines: reloading)

The continuous damage behaviour of the plaque is described by (12)–(14). As discussed in Sect. 2, the parameters for the Ogden model with the Mullins effect were calibrated against the experimental data in Maher et al. (2011). Here, the same data were used to calibrate the Ogden model with continuous damage parameters, the values of which are given in Table 3. Specifically, the second loading cycle in Maher et al. (2011) was treated as the reloading during calibration. A VUMULLINS subroutine, interfaced with Abaqus, was written for the continuous damage model. The stress–stretch response simulated using the Ogden model with continuous damage behaviour is shown in Fig. 9 for the plaque, proving a good agreement with the experimental data in Maher et al. (2011). The blue lines represent the loading response, composed of the peak stress–stretch points at a stretch level of 1.1, 1.2, 1.3, 1.4, 1.5 and 1.6, while the red lines represent the reloading response.

**Table 3:** Parameter values of Ogden model with continuous damage for plaque

*ρ* (t/mm^3^)	$\mu$ (MPa)	$\alpha$	$D$ (MPa^−1^)	*d*	$\gamma$ (mJ/mm^3^)
1.22E − 9	0.00047	20.55	0.239019	0.66	0.05

**Fig. 10:**
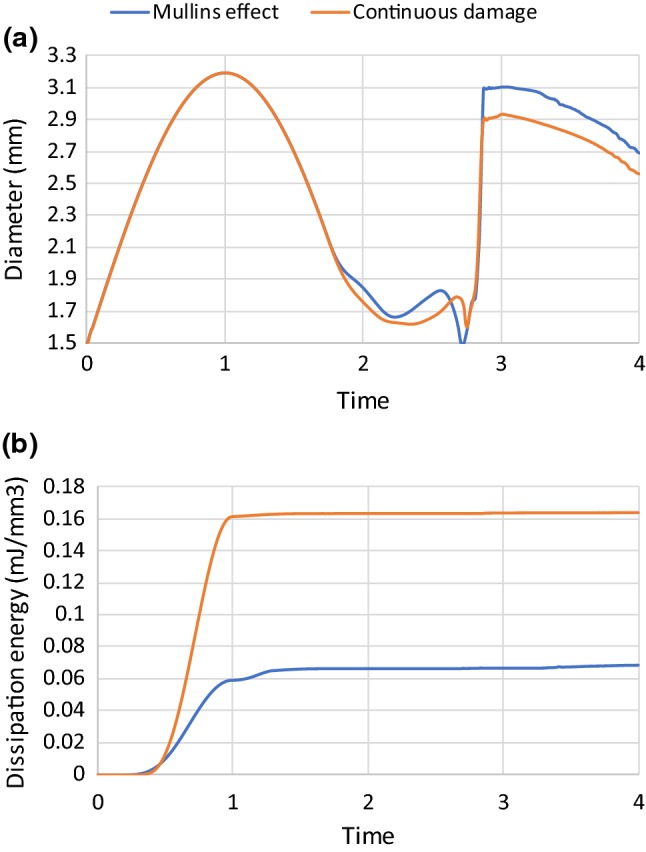
Evolutions of **a** lumen diameter and **b** dissipation energy due to damage of plaque; comparison of Mullins effect and continuous damage

The above model, capable to describe the continuous damage of the plaque, was applied to simulate the pre-dilation up to the lumen diameter of 3.2 mm followed by stenting at a pressure of 0.6 MPa. The lumen diameters achieved after pre-dilation and stenting were smaller for simulations using the continuous damage model when compared to those considering the Mullins effect only, as shown in Fig. 10a. However, the damage of the plaque was much higher for simulations considering continuous damage behaviour of the plaque, as shown in Fig. 10b. According to the theory of continuous damage, the material continuously softens in loading and unloading, so in the first few loading cycles, the plaque with continuous damage may be stiffer than that with the Mullins effect. After the artery was expanded to 3.2 mm in the lumen diameter, the damage of the plaque increased only slightly in simulations with the continuous damage model, indicating that the damage accumulated during the unloading and reloading below the primary loading did not soften the plaque much.

### Effects of plaque rupture

Another typical damage behaviour of the plaque is its rupture, which was not applied in the simulations of PCI before, to the best knowledge of the authors. In this study, the rupture of the plaque was also investigated based on a generalized damage model developed by Comellas et al. (2016). To describe the Mullins effect with rupture, the free energy function can be formulated as

15 $$ \psi = \psi_{\text{vol}} + \eta_{\text{dis}} \eta_{\text{rup}} \bar{\psi }^{0} + \phi \left( {\eta_{\text{dis}} ,\eta_{\text{rup}} } \right) $$

with

16 $$ \eta_{\text{rup}} = \left\{ {\begin{array}{*{20}l} {1,} \hfill & { \tau \le \tau^{\text{th}} } \hfill \\ {\frac{{\tau^{\text{th}} }}{\tau }\exp \left[ {{{\left( {1 - \frac{\tau }{{\tau ^{\text{th}} }}} \right)} \mathord{\left/ {\vphantom {{\left( {1 - \frac{\tau }{{\tau^{\text{th}} }}} \right)} {\left( {\frac{{g_{f} }}{{\tau^{{{\text{th}}^{2}}} }} - \frac{1}{2}} \right)}}} \right. \kern-0pt} {\left( {\frac{{g_{f} }}{{\tau ^{{{\text{th}}^{2}}} }} - \frac{1}{2}} \right)}}} \right],} \hfill & {\tau > \tau ^{\text{th}} } \hfill \\ \end{array} } \right. $$

where $\eta_{\text{rup}}$ is the rupture damage variable and $g_{f}$ is the fracture energy. The parameter $\tau$, with an initial threshold value of $\tau^{\text{th}}$, is defined as (Simo and Ju 1987)

17 $$ \begin{array}{*{20}c} {\tau = \sqrt {2\bar{\psi }^{0} } } \\ \end{array} . $$

**Fig. 11:**
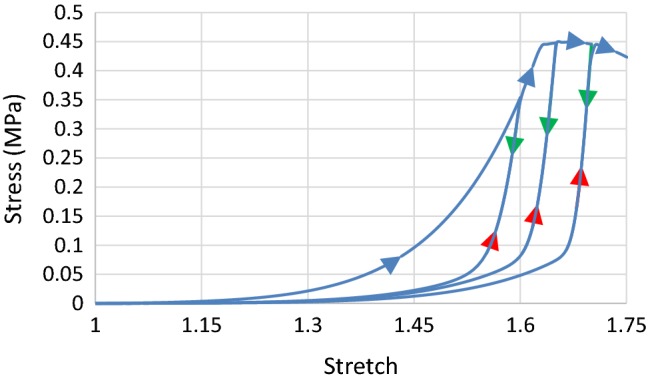
Stress–stretch responses of plaque simulated using Ogden model with both Mullins effect and rupture, where the unloading/reloading occurred at a stretch level of 1.6, 1.65 and 1.7 (Blue arrows: primary loading; green arrows: unloading; red arrows: reloading)

Here, plaque rupture is described by Eqs. (15)–(17), and the parameters for the Ogden model with the Mullin’s effect are given in Table 1. The value of $\tau^{\text{th}}$ (0.2548 N^1/2^/mm) was obtained from Cardoso and Weinbaum (2014). Due to a lack of experimental data, it was assumed that, after rupture, the material experienced low stress under further stretch, so that the value of $g_{f}$ was taken as 0.11 mJ/mm^2^. A VUMULLINS subroutine, interfaced with Abaqus, was developed for the Mullins effect model with rupture. The stress–stretch response obtained using the Ogden model with the Mullins effect and rupture is shown in Fig. 11, where the flattening portion represents the rupture (at a stretch of 1.63; blue arrows: primary loading; green arrows: unloading; red arrows: reloading).

**Fig. 12:**
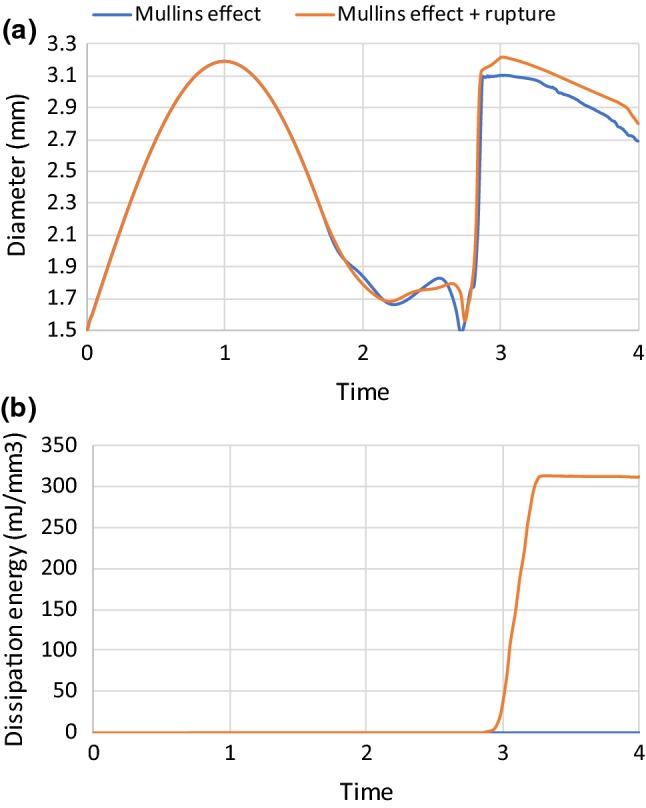
Evolution of **a** lumen diameter and **b** dissipation energy due to damage of plaque by considering plaque rupture in damage model

**Fig. 13:**
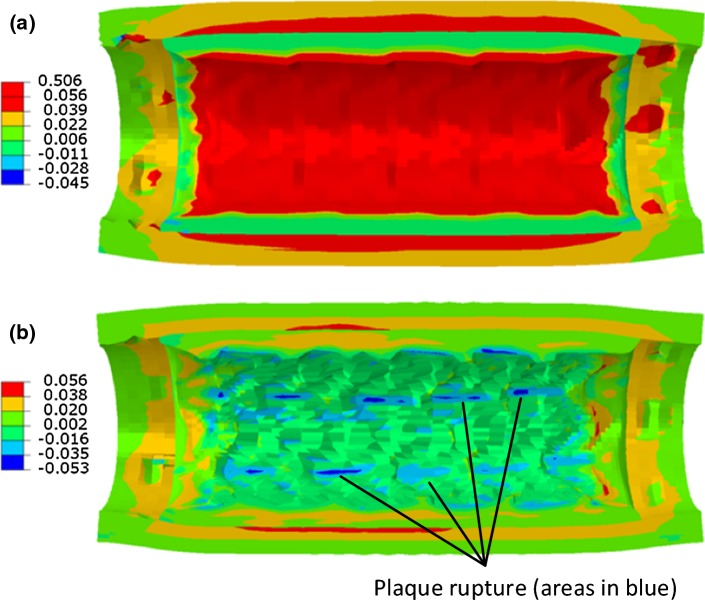
Contour plots of maximum principal stress (MPa) in artery for simulations considering **a** Mullins effect and **b** Mullins effect with rupture for plaque

The plaque rupture model described above was also applied to simulate the pre-dilation up to the lumen diameter of 3.2 mm followed by the stenting at pressure of 0.6 MPa. The results were compared to those considering the Mullins effect only (see Fig. 12). The plaque rupture led to increased expansion of the artery, with a larger lumen diameter achieved both at the peak stenting pressure and after the balloon deflation. The lumen diameter at peak stenting pressure even exceeded that in pre-dilation (i.e. 3.2 mm). For the simulations with plaque rupture, the dissipation energy due to the damage of the plaque increased considerably in the stenting procedure as shown in Fig. 12b, indicating that the occurrence of plaque rupture caused the lumen expansion. It should be noticed that the blue line in Fig. 12b is the same as the grey line in Fig. 6b, which is close to zero value but not vanishes. The rupture of the plaque was also reflected in the contour plots of maximum principal stress for plaque, as indicated in Fig. 13b (areas in blue). Basically, the inner surface of the plaque experienced high-level stresses in the simulations without considering the plaque rupture (Fig. 13a, while the stresses in the plaque nearly vanished in the analysis accounting for the plaque rupture (Fig. 13b), reflecting the loss of resistance to further stretch as result of it.

## Discussion

This study is the first attempt to investigate the effects of pre- and post-dilations on the deployment of BVS using FE simulations, with a consideration of anisotropic damage behaviour for arterial layers. The BVS currently is the most advanced type of stents with a great potential because of its capability to reduce the rates of long-term clinical complications such as late stent thrombosis, ISR and obstruction of side branches, thanks to its bioabsorbable characteristics. Although the ABSORB has been removed from the market due to acute scaffold thrombosis concerns, polymeric stents still represent the cutting edge development of next generation BVSs. The results of this investigation provide additional information to support the continuing development of BVSs. Also, pre- and post-dilations are strongly recommended for the PCI with BVSs, so it is important to understand whether and how they benefit the final outcomes of the PCI treatment. Our results showed that the pre-dilation could make it easier for the stent to expand the diseased vessel by softening its wall in advance. It is indicated that the pre-dilation should be performed, especially when the lumen area is too narrow or the plaque is heavily calcified. In addition, a larger lumen diameter could also be achieved by the post-dilation using the non-complaint balloon with higher peak pressure, resolving the situation when the stent was not fully expanded or the desired lumen diameter was not achieved. Although our investigation was focused on BVS, the results are also valid for metallic stents.

Furthermore, this study is also the first attempt to simulate the damage caused to the vessel wall by the deployment of BVS. Stenting-induced vessel damage plays a significant role in determining the severity of ISR. The recurrence of stenosis after the stent deployment is one of the major concerns regarding the PCI treatment. The mechanism of ISR is formation of neointima composed of smooth muscle cells and extracellular matrix as a result of an inflammatory reaction to the vessel damage (injury) caused by stent deployment. Our work presented the FE modelling approach to evaluate the stenting-induced damage to the layers of the vessel, which can be potentially used to assess the risk of ISR. Farb et al. (2002) reported a strong correlation between the neointimal growth and the media injury caused by stenting, which was also confirmed by Hoffmann and Mintz (2000). Essentially, the migration of smooth muscle cells from the media to the intima was stimulated by the injury to the artery during stenting. They also reported that a stent deployed with a larger diameter led to an increased area of in-stent neointima due to the increased damage of the media, as confirmed by our simulation results.

In addition, the effects of various types of plaque damage on stenting results were also studied by considering the continuous damage behaviour and the plaque rupture. The results of simulations using the former model reflected a negligible change of damage accumulation in the plaque during the deflation of pre-dilation and the following stenting procedure (i.e. unloading and reloading), suggesting that it is unnecessary to consider continuous damage behaviour in future stenting simulations. Furthermore, the larger lumen diameter was achieved, thanks to plaque rupture, suggesting the necessity of considering plaque rupture in simulations of stent deployment. However, the plaque rupture was acknowledged as a potential contributing factor to (very) late stent thrombosis (Finn and Otsuka 2012), which should be avoided or at least minimized during the stenting procedure. This rupture model can be used for predicting the risk of plaque rupture during stenting, although further experimental work should be carried out for model validation. Also, the plaques showed plastic (permanent) deformation in tensile tests (Maher et al. 2011), so further work is required to propose an improved model, capable to describe the permanent deformation of the plaque. In addition, continuous damage and rupture behaviours of arterial layers should also be explored in future studies.

According to the manufacturer’s instruction for the Absorb BVS, it is recommended that, for stent deployment, the balloon should be inflated under pressure of 2-atm (0.21 MPa) over 5 s and then deflated over a period of 30 s. However, for all simulations in this study, the step time for both the inflation and the deflation was set to be 0.1 s, i.e. significantly shorter than those recommended. This is due to the constraints on the computational time of the simulations. For instance, simulation times would be larger by a factor at least 20 for inflation and deflation of 1 s each, i.e. an increase to 10 days of simulations. This would become unrealistic for the simulations carried out. Still, the simulations for balloon inflation times of 0.1 s and 1 s were compared, and the results were within 5% difference in terms of the lumen diameter and stress distributions. In addition, it is understood that the stent deployment is a nearly static procedure, simulated as a dynamic case using Abaqus/Explicit in this study. As a result, it might cause some dynamic oscillations in the artery during the simulation. In order to reduce those dynamic effects, viscous pressure was applied to the outer surface of the artery, which was proved effective by the results obtained (i.e. no oscillation occurred in the simulations). Another limitation of this study is related to the experimental data used for damage model calibration. The data for the plaque in Maher et al. (2011) were obtained from the unconfined cyclic compression tests instead of tension, and it is worthwhile to point out that the plaque might behave differently under tension. The parameters for the MA model with damage were calibrated against the experimental data in Weisbecker et al. (2012), obtained from testing of the thoracic aorta instead of the coronary one. The fracture energy for the plaque was assumed in this study, as no experimental data are currently available. So, these limitations should be noted when interpreting the simulation results presented in this work. In addition, the artery model was idealized with a uniform and symmetric plaque layer, while, in reality, the plaques might be asymmetric, discontinuous and diffused in the lumen. Hence, patient-specific cases should be explored in the future, based on high-resolution medical imaging of actual diseased artery.

## Conclusions

The pre-dilation helped to achieve a larger lumen diameter in PCI, thanks to the softening of the plaque–artery caused by overstretch. Our numerical simulations demonstrated that the damage was always the highest in the plaque, indicating a larger possibility of plaque rupture. During the pre-dilation process, the damage of the media was higher than that of the adventitia, but the opposite case was observed for the stenting and post-dilation procedures. Again, the post-dilation helped to achieve a larger lumen diameter. When a compliant balloon was used, the highest stresses were in the artery, while for a non-compliant balloon, the highest stresses were found in the plaque, with an absence of dog-boning effect. For simulations with the continuous damage model, the damage accumulated in the unloading and reloading stages below the primary path was too small to support the following expansion of the artery, while the incorporation of the plaque rupture into the model demonstrated its positive effect on the outcome of stent deployment. Assessment of the stenting-induced damage in the arterial wall provides quantifiable physical parameters aiding the understanding of development of in-stent restenosis in patients following the stent treatment.
